# Current Status of Cell-Based Therapies for COVID-19: Evidence From Mesenchymal Stromal Cells in Sepsis and ARDS

**DOI:** 10.3389/fimmu.2021.738697

**Published:** 2021-10-01

**Authors:** Zhiheng Xu, Yongbo Huang, Jianmeng Zhou, Xiumei Deng, Weiqun He, Xiaoqing Liu, Yimin Li, Nanshan Zhong, Ling Sang

**Affiliations:** ^1^ State Key Laboratory of Respiratory Diseases, Department of Critical Care Medicine, Guangzhou Institute of Respiratory Health, First Affiliated Hospital of Guangzhou Medical University, Guangzhou, China; ^2^ Guangzhou Medical University, Guangzhou, China; ^3^ School of Public Health, Southern Medical University, Guangzhou, China; ^4^ Guangzhou Laboratory, Guangzhou, China

**Keywords:** COVID-19, MSCs therapies, ARDS, sepsis, SARS-CoV-2

## Abstract

The severe respiratory consequences of the coronavirus disease 2019 (COVID-19) pandemic have prompted the urgent need for novel therapies. Cell-based therapies, primarily using mesenchymal stromal cells (MSCs), have demonstrated safety and potential efficacy in the treatment of critical illness, particularly sepsis and acute respiratory distress syndrome (ARDS). However, there are limited preclinical data for MSCs in COVID-19. Recent studies have shown that MSCs could decrease inflammation, improve lung permeability, enhance microbe and alveolar fluid clearance, and promote lung epithelial and endothelial repair. In addition, MSC-based therapy has shown promising effects in preclinical studies and phase 1 clinical trials in sepsis and ARDS. Here, we review recent advances related to MSC-based therapy in the context of sepsis and ARDS and evaluate the potential value of MSCs as a therapeutic strategy for COVID-19.

## Introduction

**Figure 1:**
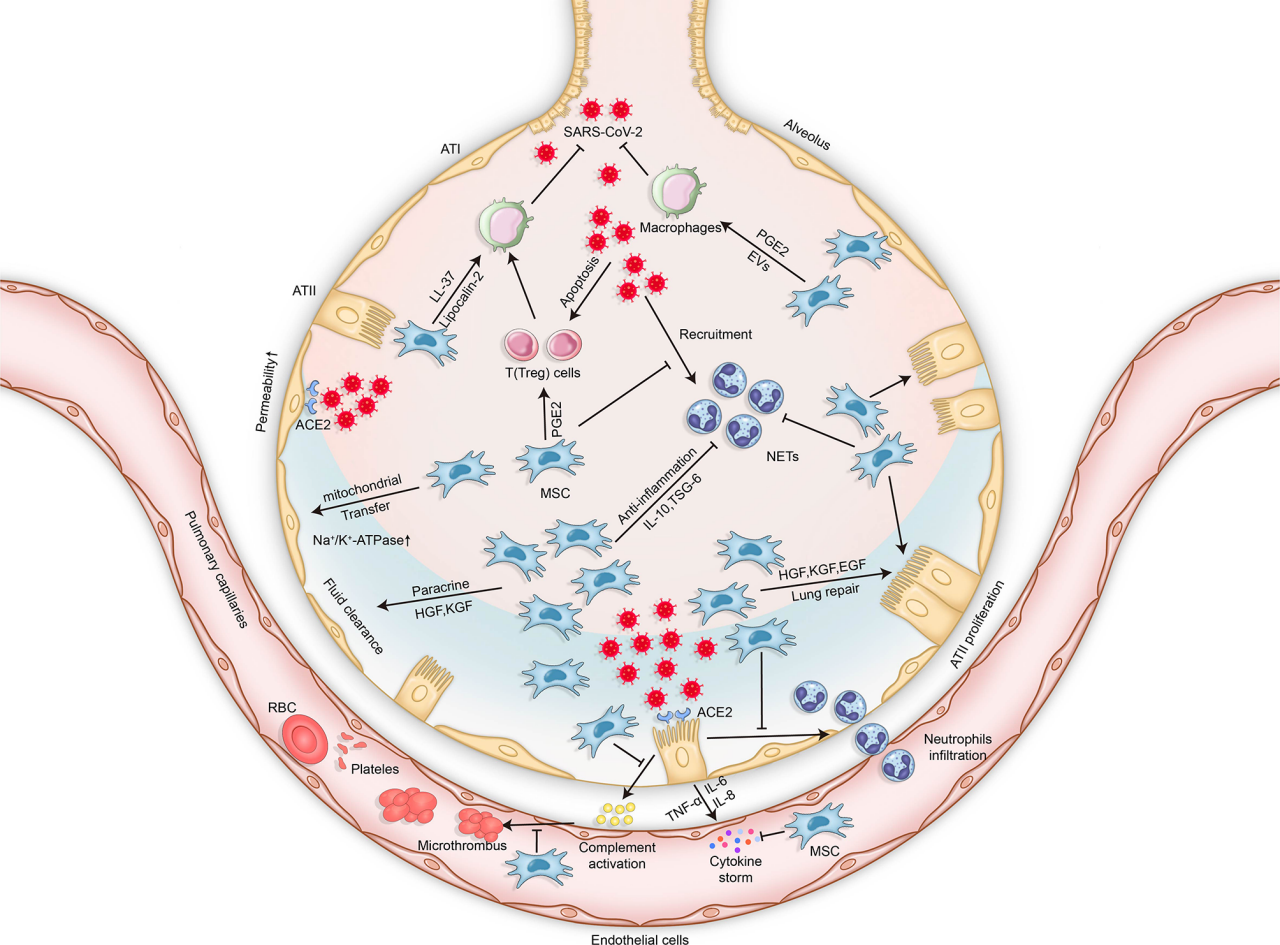
Mechanisms of lung injury in COVID-19 and potential therapeutic effects of MSCs in COVID-19-related respiratory lung injury. The mechanisms of lung injury in COVID-19 include, but are not limited to, aberrant immune host responses accompanied by a “cytokine storm,” excessive neutrophil activation, dysregulation of immune cells, endothelial dysfunction, and immunothrombosis formation. The potential therapeutic effects of MSCs in COVID-19 involve multiple mechanisms *via* their secretion of soluble paracrine factors and possible mitochondrial transfer. MSCs can enhance microbe clearance, resolve inflammation, modulate immunity, improve lung permeability, increase alveolar fluid clearance, and promote lung epithelial and endothelial repair. *SARS-CoV-2*, severe acute respiratory syndrome coronavirus 2; *MSCs*, mesenchymal stromal cells; *ATI*, alveolar type 1 cells; *ATII*, alveolar type 2 cells; *TNF*, tumor necrosis factor; *IL*, interleukin; *ACE2*, angiotensin-converting enzyme 2; *PGE2*, prostaglandin E2; *KGF*, keratinocyte growth factor; *TSG-6*, TNF-stimulated gene 6; *HGF*, hepatocyte growth factor; *EGF*, epidermal growth factor; *EVs*, extracellular vesicles; *NETs*, neutrophil extracellular traps; *RBCs*, red blood cells.

The coronavirus disease 2019 (COVID-19) pandemic, caused by severe acute respiratory syndrome coronavirus 2 (SARS-CoV-2), is rapidly and continuously spreading globally and can result in serious significant respiratory morbidity and mortality (1, 2). Although most patients with COVID-19 present with mild respiratory tract infection, severe pneumonia and acute respiratory distress syndrome (ARDS) have been described in 19% of reported cases, and the overall mortality is approximately 49% (3). The most common symptoms of SARS-CoV-2 infection are cough, fever, fatigue, headache, myalgia, and diarrhea. Approximately 1 week after the onset of symptoms, patients become severely ill when dyspnea and hypoxemia appear, and progressive respiratory failure subsequently develops (4). The main reason for severe COVID-19 disease is aberrant immune host response accompanied by a “cytokine storm.” More precisely, it involves the excessive activation and dysregulation of CD8^+^ T cells and immoderate pulmonary recruitment of immune cells, as well as the overproduction of pro-inflammatory cytokines such as interleukin 2 (IL-2), IL-6, IL-7, and tumor necrosis factor (TNF) (5). Additionally, endothelial dysfunction and immunothrombosis are also regarded as critical pathogenic mechanisms leading to severe COVID-19 (6) (**Figure 1**).

Without effective antiviral medications, current therapeutic approaches are limited to aggressive standard supportive care and treatment of any other co-infections. More recently, a growing number of clinical investigations of cell-based therapies, primarily involving mesenchymal stromal cells (MSCs), have demonstrated safety and possible efficacy in the treatment of critical illness, particularly in sepsis and ARDS (7–15). Recent preclinical data in models of sepsis/ARDS and relevant related clinical studies of MSC administration in patients with ARDS may contribute to a better understanding of the potential MSC-based cell therapy approaches for COVID-19. Therefore, our review focused on MSCs as a potential therapeutic agent for COVID-19 on the basis of the current evidence from sepsis and ARDS.

## Mesenchymal Stromal Cells

MSCs were originally characterized in bone marrow stromal cells and are now the best-described and most widely used cells for cell-based therapies. According to the International Society of Cellular Therapy, MSCs are defined by the following minimal criteria: 1) must be adherent to plastic; 2) must positively present CD105, CD90, and CD73 and must be lacking CD45, CD34, CD14, CD11b, CD79 alpha, CD19, and HLA-DR surface makers; and 3) must have a differentiation potential in *in vitro* conditions (16).

## Sources of MSCs

MSCs were originally identified in the bone marrow, and the most recent studies have focused on the effects of bone marrow-derived MSCs. However, MSCs can also be isolated from several tissues such as umbilical cord/fetal blood, placenta, amniotic fluid, and adipose tissue (17–21). Recent studies on rodent models have shown that adipose tissue-derived MSCs minimized ischemia–reperfusion lung injury by suppressing oxidative stress and inflammatory reaction (22); improved phagocytosis and the bactericidal functions of macrophages in a model of *Pseudomonas aeruginosa* pneumonia (23); attenuated radiation-induced lung injury through anti-inflammation, anti-fibrosis, and anti-apoptosis mechanisms (24); and abrogated bleomycin-induced lung fibrosis by significantly reducing collagen deposition at an early stage (25). Moreover, it was also reported that umbilical cord-derived MSCs can ameliorate hyperoxia-induced lung injury in newborn rats with bronchopulmonary dysplasia and improve the bacterial clearance and survival in neonatal septic rats (26, 27).

## Attractive Therapeutic Candidates Based on MSCs

There are many advantages of MSCs as an attractive therapeutic candidate. Firstly, they express low levels of human leukocyte antigen (HLA)-associated proteins, which lead to the absence of immunosuppression by allogeneic MSCs as stimulators (28). Secondly, MSCs can be easily isolated from host tissue and expanded stably and rapidly *in vitro*, which enables sufficient quantities for clinical use (29). Thirdly, the homing capacity of MSCs to the injury sites contributes to their persistence in the target tissues (30). Moreover, MSCs can be trapped in the lungs by systemic administration, thus prolonging their persistence in the lungs, which may be of most benefit in lung disease treatment (30). Fourthly, the safety profile of MSCs was demonstrated by phase 1 clinical trials in patients with ARDS and sepsis (7–15). Finally, growing evidence shows that MSCs exert anti-inflammatory and anti-apoptotic effects in response to injury (31).

## Therapeutic Mechanisms of MSCs

### Paracrine Cytokines

Paracrine secretion is a well-known mechanism for MSCs to exhibit their potential benefits (32). A paracrine mechanism of the small mediators secreted by MSCs was suggested to exert the effects of immune modulation, inflammation resolution, and injury restoration. Subsequent studies revealed that several paracrine factors are involved in MSC secretomes, including LL-37 (antimicrobial peptides) (33), keratinocyte growth factor (KGF, enhanced alveolar fluid clearance) (34, 35), angiopoietin-1 (Ang-1, restored epithelial cell permeability) (36), hepatocyte growth factor (HGF, restored endothelial cell permeability) (37–39), and lipoxin A4 (LXA4, alleviated inflammation) (36). For example, in an *in vitro* experiment, conditioned medium (CM) from *Escherichia coli*-stimulated MSCs, compared with unstimulated MSCs, showed remarkable antimicrobial activity against *E. coli* and *P. aeruginosa*, followed by a significantly higher level of LL-37 being observed in MSCs after bacterial challenge. However, *E. coli*-stimulated MSCs pre-incubated with the anti-LL-37 antibody resulted in a decrease in bacterial clearance. A subsequent *in vivo* experiment also displayed a consistent result, suggesting that the antimicrobial activity of MSCs is mainly attributed to the secretion of the antimicrobial peptide LL-37 (33). In addition, a study by Wang et al. (38) reported that the ability of bone marrow-derived MSCs to stabilize the endothelial barrier was partly affected by HGF. Lipopolysaccharide (LPS)-induced endothelial paracellular and transcellular permeabilities were improved after treatment with MSC macrovesicles (MSC-MVs), accompanied by more expressions of the endothelial intercellular junction proteins VE-cadherin and occludin and less endothelial apoptosis. However, these effects were removed after HGF gene knockdown in MSC-MVs. Carrying plenty of cytokines with specific function, MSCs were shown to be of great benefit to the resolution of inflammation and the repair of acute lung injury (ALI).

### Mitochondrial Transfer

Mitochondrial transfer is also considered as one of the important mechanisms underlying the protective effects of MSCs (40). MSCs can aid the repair of lung injury through the transfer of mitochondria, which results in increased alveolar ATP concentration (41). Moreover, mitochondrial transfer from MSCs to alveolar macrophages leads to the enhancement of phagocytic activity, thus exerting antimicrobial effects in ARDS (42). Additionally, MSCs can promote an anti-inflammatory and phagocytic macrophage phenotype *via* extracellular vesicle-mediated mitochondrial transfer (43). Finally, MSCs can donate cytoplasmic content and mitochondria to repair damaged bronchial epithelial cells (44). However, the microenvironment is crucial for effective MSC therapy. It was reported that hypercapnic acidosis induces mitochondrial dysfunction and impairs the ability of MSCs to promote distal lung epithelial repair (45, 46), which may lead to the invalidation of MSCs in patients who develop hypercapnia. Another study further illustrated that the administration of MSC extracellular vesicles with dysfunctional mitochondria had no effect in attenuating LPS-induced ALI, whereas normal MSC extracellular vesicles were able to restore mitochondrial respiration and improve barrier integrity (47).

## MSCs in Sepsis Treatment

In COVID-19 patients who develop acute respiratory failure requiring intubation and mechanical ventilation, sepsis and sepsis shock can also develop. Sepsis is a systemic inflammatory response to infection that is characterized by the imbalance between pro-inflammation and anti-inflammation (48, 49). Recently, MSCs have been shown to be effective in modulating such processes, which may be applicable to COVID-19.

### Enhancement of Microbe Clearance

#### Antibacterial Effect of MSCs

Sepsis is a multifaceted host response to an infecting pathogen. The clearance of bacteria with antibiotics is one of the most important treatments for sepsis (50, 51). Beyond antibiotics, MSCs were recently reported to play a crucial role in clearing bacteria in sepsis (52, 53). In *in vivo* experimental models of sepsis, MSC delivery was shown to be significantly greater in the clearance of bacteria *via* enhancing the phagocytotic activity of neutrophils, monocytes, and macrophages (54–56). In addition, MSCs could secrete antimicrobial factors such as LL-37 (33, 57) and lipocalin 2 (53) to reduce bacterial growth (in colony forming units). This bactericidal effect may be the result of the enhancement of NOX2-dependent reactive oxygen species (ROS) production by MSCs (55). Furthermore, MSC extracellular vesicles suppressed the activity of multidrug resistance-associated protein 1 (MRP1) through the transfer of *miR-145*, thereby resulting in antimicrobial activity in *E. coli*-induced pneumonia in C57BL/6 mice through LTB4/BLT1 signaling (58).

#### Antiviral Effect of MSCs

Aside from bacterial infection in sepsis, viral infection is an independent risk factor for sepsis and septic shock (59, 60). However, the antiviral effect of MSCs remains controversial in preclinical studies. MSCs are susceptible to H1N1, H5N1, and H9N5 avian influenza virus, which may lead to cell death and induce the immune dysregulation of MSCs by releasing high levels of TNF-α, IL-6, MCP-1, and MIP-1β (61–63). Moreover, MSC therapy was shown to prolong the disruption of the alveolar–capillary barrier and failed to improve outcomes in experimental severe influenza in mice (64, 65). In contrast, more recent studies of MSCs have reported their protective effects in influenza virus-induced ALI in mice (66–69). The inconsistent results on the antiviral effects of MSCs may have been dependent on the different types of influenza virus and MSCs, animal models, ages, and the methods of administration. A study by Michael et al. showed that MSC treatment was beneficial in aged mice (8–12 months of age), but not in young mice (6–8 weeks of age) with H5N1 influenza virus infection (66). To avoid infection of MSCs by the influenza virus, Mahesh et al. applied MSCs derived from extracellular vesicles for the treatment of influenza-infected pigs, which showed great benefits in attenuating ALI and may provide a cell-free therapeutic strategy for influenza in humans (68). Loy et al. reported that umbilical cord MSCs (UC-MSCs) with greater growth factor secretion of Ang-1 and HGF outperformed bone marrow-derived MSCs in restoring the impaired alveolar fluid clearance and protein permeability of H5N1-infected alveolar epithelial cells (69). Although SARS-CoV-2, a coronavirus, may differ from influenza virus, the use of MSCs in COVID-19 should be fully considered according to the double-edged sword of MSC application in influenza.

#### Antifungal Effect of MSCs

Fungal causes of sepsis have increased rapidly worldwide (59, 70). However, there remains a lack of information regarding MSC treatment in fungal infection. It was reported that a subset of single colony-derived MSCs producing IL-17 was capable of inhibiting the growth of *Candida albicans* both *in vitro* and *in vivo* (71). Thus, the potential antifungal effect of MSCs for sepsis and ALI warrants rapid investigation.

### Resolution of Inflammation

The reduction and resolution of inflammation is of vital importance in sepsis. MSCs seem to be promising in this respect. It was reported that MSC administration contributed to a reduction in the pro-inflammatory responses (TNF-α, MIP-2, and IL-6) to endotoxins while upregulating the anti-inflammatory cytokine IL-10 (72–78). A network analysis of transcriptional responses showed that more than 4,000 genes were significantly altered in MSC-treated mice, which indicated that the protective effect of MSCs in sepsis was not limited to a single mediator (79). Of these, TNF-α stimulated gene 6 (TSG-6) may serve as a potential biomarker to predict the efficacy of MSCs in modulating inflammation because MSCs with high levels of TSG-6 exert an enhanced anti-inflammatory efficacy (80–82). However, MSCs are highly sensitive to their microenvironment (46). Under excessive inflammatory circumstances, they may undergo apoptosis through inflammation-induced autophagy (83). Conversely, MSC-conditioned media with an anti-IL6 antibody was more effective in augmenting the promotion of an anti-inflammatory monocyte phenotype (84). In addition, MSCs could restrict NLRP3 inflammasome activation, which suppressed the generation of mitochondrial ROS (85). Moreover, MSC-conditioned media contributed to a reduction in the activity of NF-kB and MMP-9 in neutrophils from patients with sepsis-related ARDS, thus exhibiting great potential in the treatment of inflammatory disorders (86).

### Modulation of Immunity

Immunological dysfunction is also an important clinical manifestation of sepsis and septic shock (87, 88). MSCs were shown to possess immunomodulatory functions in sepsis. MSCs expressing toll-like receptor 4 (TLR4) and myeloid differentiation primary response gene-88 (MyD88) are essential for releasing prostaglandin E2 (PGE_2_), which can reprogram macrophages to increase the production of IL-10 (89, 90). Along with the prostaglandin E2 receptor, PGE_2_ is indispensable to optimal CD4^+^ T-cell activation and T-cell-mediated inflammatory responses (91). When PGE_2_ synthesis of human MSCs was inhibited, the MSC-mediated immunosuppressive effects on T cells were also negated (92). In this respect, MSCs may be useful in COVID-19 management where aberrant immune activation plays an important role in the disease progression (5). Moreover, circulating CD3^+^CD4^+^CD25^+^ regulatory T cells (Tregs) were significantly increased when MSCs were administered in a cecal ligation and puncture (CLP)-induced sepsis rat model (93). Furthermore, the immunosuppressive capacity of Tregs can inhibit the production of pro-inflammatory cytokines such as IL-6 and TNF-α (93, 94). In addition, MSCs exhibited immunosuppressive effects by reducing the level of immunoglobulin production and chemokines that are related to recruiting neutrophils in lung B cells (95). When IL-1β was pretreated, the immunomodulatory properties of MSCs could be effectively enhanced (96). MSC-mediated immunomodulation during sepsis was likely to be associated with the MyD88–NFκB signaling pathway (74).

## MSCs in ARDS Treatment

Characterized by the acute onset of non-cardiogenic pulmonary edema, hypoxemia, and diffuse alveolar–capillary membrane damage, ARDS is a common cause of respiratory failure in critically ill patients (97). Recently, a growing number of studies have suggested that MSCs may be a potential therapy for ARDS by improving lung permeability, increasing alveolar fluid clearance, and promoting lung epithelial and endothelial repair.

### Improvement of Lung Permeability

Epithelial injury is critical in the development of ALI. Alveolar epithelial cells may be the first to be affected by pulmonary ARDS (caused by pneumonia, acid aspiration, and toxic gas inhalation), which may contribute to the loss of the alveolar epithelial barrier integrity and thus increase lung permeability (98). MSCs were reported to show benefits in improving lung epithelial permeability, such as restoring the permeability of alveolar type II epithelial cells through the secretion of Ang-1 (36). Additionally, sodium transport and epithelial permeability were recovered by conditioned media from MSCs *via* the secretion of KGF in an *in vitro* model of acute alveolar injury (99), while the intrapulmonary delivery of MSCs contributed to significantly decreasing the levels of bronchoalveolar lavage protein and improving the alveolar epithelial permeability in endotoxin-induced ALI in mice (72). The improvement in alveolar epithelial permeability by MSCs may be a result of the activation of Wnt3a-induced Wnt/β-catenin signaling (100).

Similarly, pulmonary vascular endothelial cells may be the first to be injured by extrapulmonary ARDS (resulting from sepsis, trauma, or hemorrhagic shock), which may lead to dysfunction of the pulmonary vascular endothelial barrier and, thus, increase lung permeability (101). It was reported that MSCs inhibit pulmonary vascular endothelial permeability by preserving critical vascular endothelial barrier proteins (VE-cadherin, occludin-1, and claudin-1) in the lungs after hemorrhagic shock (102, 103). Adipose-derived stem cells also attenuated pulmonary microvascular hyperpermeability in a sheep model of smoke inhalation-induced ALI (104). MSC treatment for endotoxin-induced lung injury improves the lung endothelial barrier permeability partly by secreting KGF and HGF (37, 38, 105). The capacity of MSCs to inhibit vascular permeability is likely through the modulation of VE-cadherin/β-catenin signaling (106).

### Increment of Alveolar Fluid Clearance

Alveolar fluid clearance is usually impaired in patients with ARDS (107) and contributes to lung edema and hypoxemia. MSC treatments were reported to play a significant role in clearing alveolar fluid. In an *in vivo* mouse model of *E. coli* endotoxin-induced ALI, bone marrow-derived MSCs were shown to reduce extravascular lung water by 60% and total protein levels by 66% (108). In a sheep model of ALI, MSCs also attenuated the lung water content and edema (104, 109). In an *ex vivo* perfused human lung endotoxin-induced ALI model, MSCs increased the alveolar fluid clearance partly by restoring sodium transport (105). The above benefits of MSC treatments may have originated from the paracrine signaling of KGF, which contributed to restoring the effects of the α-epithelial sodium channel (α-ENaC) (99, 110, 111).

### Promotion of Lung Epithelial and Endothelial Repair

The promotion of lung epithelial and endothelial cell repair is of crucial importance in ARDS treatment. MSC-based therapy may be the breaking point in lung repair and regeneration. MSC therapy enhances pulmonary epithelial wound healing and restores lung structure by secreting KGF in ventilator-induced lung injury (34). MSCs can also improve the viability of BEAS-2B cells and inhibit LPS-induced apoptosis, which may be due to the increased expression of proliferating cell nuclear antigen (PCNA) and KGF and the reduced expression of caspase-3 (112). In addition, receptor tyrosine kinase-like orphan receptor 2 (ROR2)- (113), β-catenin- (114), and KGF-overexpressing (115) MSCs significantly promoted their proliferation and differentiation into type II alveolar epithelial cells, which demonstrated much better therapeutic effects than did native MSCs alone. Furthermore, MSCs can mitigate LPS-induced injury in murine lung epithelial cells and reverse the epithelial–mesenchymal transition by blocking the activation of the NF-κB and Hedgehog pathways (116). Finally, the repair and regeneration potential of MSCs may be induced by the stimulation of pro-inflammatory cytokines (i.e., TNF-α and IL-1β) and the activation of Jun N-terminal kinase (JNK) and p38 mitogen-activated protein kinase (MAPK) (117, 118).

## MSCs in COVID-19-Related ARDS

According to statistical data generated early in the COVID-19 pandemic, 14% of cases were classified as severe COVID-19 and 5% were determined as critical with organ failure (3). Severe pneumonia and ARDS have been described in the reported COVID-19 cases. In parallel with proper ventilation management, dexamethasone, antiviral agents, convalescent plasma, and IL-1/6 inhibitors, MSCs are also considered a promising candidate therapy for COVID-19-related ARDS (4, 119). It was reported that MSCs from the bone marrow, amniotic fluid, and adipose tissue were all resistant to SARS-CoV-2 infection due to the low expressions of angiotensin-converting enzyme 2 (ACE-2) and transmembrane protease serine subtype 2 (TMPRSS2) on the cell surface under both steady-state and inflammatory conditions (120, 121), which supported the potential applicability of MSCs for COVID-19 treatment (**Figure 1**).

It was reported that MSCs were well tolerated in ARDS patients without severe adverse events in phase 1 and phase 2a clinical trials (13, 15). Even though adverse effects such as shivering, muscle contraction, and increased lactic acid dehydrogenase arose after the infusion of MSCs in a small minority of patients (122–124), multiple clinical studies have identified the tolerability and efficacy of MSCs in the treatment of COVID-19-related ARDS. Current clinical trials advise that it is safe to administer MSCs extracted from the umbilical cord, placenta, or menstrual blood to severely and critically ill COVID-19 patients (122–132), always intravenously, with one to three infusions and 10^6^–10^8^ cells per dose.

Leng et al. (128) reported the earliest clinical trial (ChiCTR2000029990) of MSC therapy against COVID-19. In one dose of 1 × 10^6^ cells/kg, clinical grade MSCs were intravenously transplanted to seven patients with COVID-19 pneumonia (one of the critical type, four of the severe type, and two of the common type), and three patients in the control group received placebo treatment. Without any observed adverse effects, the symptoms (high fever, weakness, and shortness of breath) and oxygen saturations significantly improved within 2–4 days after MSC transplantation in all patients. More importantly, it was suggested that MSCs were valuable in suppressing virus-induced cytokine storms. Compared with the placebo control group, the overactivated cytokine-secreting immune cells disappeared in 3–6 days and regulatory dendritic cells dramatically increased in the group that received MSC treatment. Meanwhile, the serum pro-inflammatory cytokine TNF-α was significantly decreased and the anti-inflammatory cytokine IL-10 was remarkably increased after MSC transplantation. Of note is that all seven patients were put forward for this pilot study after no improvements were observed under standard treatment, and thus, it is not surprising that patients in the placebo group showed negative results. Therefore, the functions of MSCs should be identified in a larger population.

In a multicenter, open-label, non-randomized, parallel controlled phase 1 exploratory trial in China (ChiCTR2000029606) (125), 44 severe or critical COVID-19 patients were enrolled. Among them, 26 patients received allogeneic, menstrual blood-derived MSC therapy (three infusions totaling 9 × 10^7^ MSCs, one infusion every other day), while 18 patients received only concomitant medication. After 1 month of follow-up, it was observed that the mortality between the two groups was significantly different (7.69% in the MSC group *vs*. 33.33% in the control group, *p* = 0.048). Compared with the control group, the symptoms of patients in the experimental group were quickly alleviated, with expiratory dyspnea significantly improving during MSC infusion on day 1 (*p* = 0.016), day 3 (*p* = 0.040), and day 5 (*p* = 0.031). Chest imaging results also showed improvements in the experimental group in the first month after MSC infusion. In addition, Lanzoni et al. (132) reported a double-blind, phase 1/2a, randomized controlled trial of UC-MSC therapy against COVID-19. A total of 24 patients who were diagnosed with COVID-19-related ARDS within 24 h were randomized 1:1 to either a UC-MSC treatment group (two intravenous infusions of 100 ± 20 × 10^6^ UC-MSCs, on days 0 and 3) or a control group (two infusions of vehicle solution). The results reflected that UC-MSC treatment resulted in significant reductions of inflammatory cytokines on day 6 and improved survival (91% *vs*. 42%, *p* = 0.015) during a 31-day follow-up. In another double-blind, multicenter, phase 1 randomized controlled trial (NCT04457609) (130) in 40 critically ill COVID-19 patients, 20 patients received a single intravenous infusion of 1 × 10^6^/kg UC-MSCs in 100 ml saline (0.9%) solution (SS) and 20 patients received 100 ml 0.9% SS as the control group. The median time from intensive care unit (ICU) enrollment to MSC treatment was 8 days (range = 2–30 days). During 30 days of observation after MSC administration, the results revealed that UC-MSC treatment was significantly associated with a higher survival rate, with particular benefit in patients with comorbidities. In conclusion, MSC therapy is a safe approach against COVID-19 disease and shows promising benefits for severely ill COVID-19 patients at a progression stage. However, the observation periods of these studies were short and the sample size was small; thus, whether MSC therapy has long-term benefits warrants further research.

In a study (NCT04288102) by Shi et al. (131), a randomized, double-blind, placebo-controlled phase 2 trial was performed in 100 severe COVID-19 patients who were randomly assigned to receive either UC-MSCs (4 × 10^7^ cells per infusion) or placebo on days 0, 3, and 6. According to the analyses of both radiologists and lung imaging artificial intelligence software, the results of high-resolution chest CT imaging revealed that the proportions of solid component lesion volumes were significantly reduced in the MSC treatment group, but not in the placebo group, after 28 days of follow-up. The results of a 6-min walk test also indicated better restoration in patients treated with UC-MSCs. However, with a median time from symptom onset to study baseline of approximately 45 days, most of these patients were at the convalescent stage when joining this trial, which might account for the lack of significant reduction in the duration of oxygen therapy, dyspnea scores, cytokine levels, or chemokine levels. Nonetheless, COVID-19 patients might benefit from MSC therapy, even in the convalescent stage.

Several clinical studies were performed to assess the safety and effectiveness of MSCs in COVID-19 disease (**Table 1**). However, these preclinical trials seemed to be less convincing due to the small sample sizes ranging from 5 to 100. Because of the emergency nature of the COVID-19 outbreak and the ethical limitations, some of these studies failed to strictly follow the principles of standard clinical trials, such as randomization, blindness, and comparisons. In addition, the patients enrolled in these clinical studies were at different stages of severe COVID-19 disease, which resulted in greater heterogeneity and different results. In particular, given that intravascular coagulation and thromboembolism are considered as leading causes of fatality in COVID-19, the application of MSCs remains controversial because variable levels of highly procoagulant tissue factor (TF/CD142) are expressed by MSCs (133). Furthermore, the time to starting MSC treatment, the cell dosage, and the interval duration have not been fully determined. Hence, further high-quality randomized clinical trials are needed to provide evidence regarding the application of MSCs in COVID-19-related ARDS, and hundreds of clinical trials registered on (https://clinicaltrials.gov/) may offer assistance with this.

**Table 1:** Clinical trials of mesenchymal stromal cells (MSCs) in acute respiratory distress syndrome (ARDS) and sepsis.

First author (reference)	Journal (publication year)	Study design	MSC (*n*)	Control (*n*)	MSC source	Dose of MSC	Main outcome
**MSC therapy in sepsis**							
Perlee (10)	Stem Cells (2018)	Single-blind phase 1 RCT	24	8	Adipose tissue	Dose escalation: 1/5/10 × 10^6^ cells/kg	ASCs are safe and exerted a variety of time-dependent pro-inflammatory and anti-inflammatory effects, as well as procoagulant features.
McIntyre (11)	Am J Respir Crit Care Med (2018)	Open-label phase 1 clinical trail	9	21	Bone marrow	Dose escalation: 0.3/1/3 × 10^6^ cells/kg	BM-MSCs are safe for patients with septic shock and appeared to attenuate the levels of several pro-inflammatory cytokines.
He (12)	Transl Res (2018)	Open-label phase 1 clinical trail	15	15	Umbilical cord	Dose escalation: 0.25/1/4 × 10^6^ cells/kg	A single intravenous MSC infusion of up to 3 × 10^6^ cells/kg was well tolerated and is safe in patients with severe sepsis.
**MSC therapy in ARDS**							
Zheng (14)	Respir Res (2014)	Double-bind phase 1 RCT	6	6	Adipose tissue	1 × 10^6^ cells/kg IBW	Safe and feasible. The length of hospital stay, ventilator-free days, and ICU-free days at 28 days were similar between groups.
Wilson (13)	Lancet Respir Med (2015)	Open-label phase 1 clinical trial	9	0	Bone marrow	Dose escalation: 1/5/10 × 10^6^ cells/kg IBW	An infusion of up to 10 million cells/kg IBW was well tolerated without serious adverse events.
Matthay (15)	Lancet Respir Med (2019)	Double-blind randomized phase 2a RCT	40	20	Bone marrow	1 × 10^7^ cells/kg IBW	Patients in the MSC group had numerically higher disease severity scores than those in the placebo group at baseline, but mortality did not differ significantly between groups.
Chen (8)	Engineering (Beijing) (2020)	Open-label clinical trail	17	44	Menstrual blood	100 ml	MSCs significantly improved the survival rate of H7N9-induced ARDS.
Yip (7)	Crit Care Med (2020)	Prospective phase 1 clinical trial	9	0	Umbilical cord	Dose escalation: 1/5/10 × 10^6^ cells/kg	No serious adverse events were identified in any patient, and circulating inflammatory biomarkers were notably progressively reduced.
**MSC therapy in COVID-19-associated ARDS**							
Leng (128)	Aging Dis (2020)	Open-label non-randomized pilot trial	7	3	Not applicable	One dose of 1 × 10^6^/kg	MSCs could cure or significantly improve the functional outcomes of seven patients without observed adverse effects.
Shu (124)	Stem Cell Res Ther (2020)	Open-label controlled cohort study	12	29	Umbilical cord	2 × 10^6^ cells/kg IBW	MSC treatment is safe and effective at preventing deterioration in severe COVID-19.
Sengupta (129)	Stem Cells Dev (2020)	Prospective non-randomized open-label cohort study	25	2	Exosomes derived from BM-MSCs	A total of 15 ml exosomes	MSC exosomes exhibited capacity to restore oxygenation, downregulate cytokine storm, and reconstitute immunity.
Meng (126)	Signal Transduct Target Ther (2020)	Controlled non-randomized phase 1 clinical trial	9	9	Umbilical cord	Three infusions of 3 × 10^7^ MSCs	Intravenous MSC infusion in patients with moderate and severe COVID-19 is safe and well tolerated.
Feng (127)	Cell Prolif (2020)	Single-arm pilot trial	16	0	Umbilical cord	Four infusions of 1 × 10^8^ MSCs	Intravenous transplantation of UC-MSCs is safe and feasible for the treatment of patients with severe and critically severe COVID-19 pneumonia.
Dilogo (130)	Stem Cells Transl Med (2021)	Multicenter double-blind RCT	20	20	Umbilical cord	1 × 10^6^ cells/kg	Intravenous infusion MSCs increased the survival rate by modulating the immune system toward an anti-inflammatory state.
Shi (131)	Signal Transduct Target Ther (2021)	Double-blind phase 2 RCT	65	35	Umbilical cord	Three infusions of 4 × 10^7^ MSCs	UC-MSC administration is safe and accelerated the resolution of lung solid component lesions and improvement in the integrated reserve capability.
Xu (125)	Clin Transl Med (2021)	Multicenter open-label non-randomized trial	26	18	Menstrual blood	Three infusions of 3 × 10^7^ MSCs	Patients in the MSC group showed significantly lower mortality and improvements in dyspnea, SpO_2_, and chest images.
Lanzoni (132)	Stem Cells Transl Med (2021)	Double-blind phase 1/2a RCT	12	12	Umbilical cord	Two infusions of 1 × 10^8^ MSCs	Inflammatory cytokines were significantly decreased in MSC-treated subjects, and treatment was associated with significantly improved patient survival.
Iglesias (122)	Aging Dis (2021)	Open-label longitudinal pilot trial	5	0	Umbilical cord	l × l0^6^ cells/kg	Three patients survived and were extubated on the ninth day post-infusion. Two patients died 13 and 15 days after infusion.
Hashemian (123)	Stem Cell Res Ther (2021)	Open-label two-center phase 1 single-arm trial	11	0	Umbilical cord; placenta	Three infusions of 2 × 10^8^ MSCs	Treatment of MSCs could rapidly improve respiratory distress and reduce inflammatory biomarkers.

Treatments in the control arm are not shown because they are always mentioned as “placebo” in the original text.

RCT, randomized controlled trial; IBW, ideal body weight; BM-MSCs, bone marrow-derived mesenchymal stromal cells; ASCs, allogeneic adipose MSCs.

## Gene Engineering Studies of MSCs

The homing capacity and the systemic distribution of MSCs contribute to the persistence of MSCs in the lungs (26). Moreover, interesting work has been undertaken on therapeutic factors, such as Ang-1, KGF, and HGF, in ARDS and sepsis. However, MSCs alone or “protected mediators” alone may not be sufficient to rectify the disorder of the microenvironment, particularly in severe ARDS and septic shock. Therefore, there is a strong rationale for gene-modified MSCs with critical molecules to “rescue” lung injury and sepsis. It was reported that Ang-1-transfected MSCs could further improve both alveolar inflammation and permeability in septic mice (134, 135). In addition, ROR2-overexpressing MSCs led to more significant effects than did the native MSC treatment in ARDS mice, including the retention of MSCs in the lung, differentiation into alveolar type 2 (ATII) cells, and promotion of lung repair (113). Moreover, gene-modified MSCs also showed further benefits in LPS-induced sepsis and ALI treatment than did native MSCs alone, including overexpressing β-catenin, KGF, HGF, IL-10, the E-prostanoid 2 receptor, CXCR4 receptor, soluble IL-1 receptor-like-1 (sST2), ACE2, TGFβ1, CXCR7, LL-37, heme oxygenase-1, angiotensin II type 2 receptor (AT2R), Miro1, and 7ND (dominant-negative inhibitor of CCL2) (114, 115, 136–148) (**Table 2**). Aside from the above-mentioned coding genes, the modification of non-coding genes, microRNAs, is an alternative approach. For instance, Wei et al. (150) reported that miR-377-3p released by UC-MSCs suppressed the expression of the target gene *RPTOR*, and *RPTOR* silencing played a pivotal role in enhancing autophagy-related proteins. Administration of UC-MSCs with overexpression of miR-377-3p activated the process of autophagy in alveolar epithelial cells under LPS treatment and finally dramatically ameliorated inflammation both *in vitro* and *in vivo*. Besides, miR-124-3p transferred by MSC-derived exosomes was observed to improve oxidative stress injury and suppress inflammatory response, thus was beneficial to attenuate traumatic ALI in rats (151). It was also reported that the transduction of miRNA *let-7d* (with anti-fibrotic effects) promoted recovery from bleomycin-associated lung injury (149). However, there are no reports on which genes should be overexpressed or knocked out in different models of sepsis and ARDS, and it is not clear whether double or triple knockouts of specific genes overexpressed in MSCs are better than a single gene. There is also no report of whether genetically modified MSCs are safe or effective for use in the human body. Therefore, further preclinical studies are required to elucidate the exact role of gene-modified MSCs in different microenvironments.

**Table 2:** Genetically modified MSCs in treatment-based studies using models of ALI and sepsis.

Gene/RNA	Model	MSC source	Dose	Administration	Reference
ROR2	LPS-induced ALI	Bone marrow from mice	5 × 10^5^ cells	i.t.	(113)
β-catenin	LPS-induced ALI	Bone marrow from mice	5 × 10^5^ cells	i.t.	(114)
KGF	LPS-induced ALI	Bone marrow from mice	5 × 10^5^ cells	i.v.	(115)
ANGPT1	LPS-induced ALI	Murine MSCs	2.5 × 10^5^ cells	i.v.	(135)
IL-10	LPS-induced ALI	Bone marrow from mice	1 × 10^6^ cells	i.t.	(136)
EP2	LPS-induced ALI	Bone marrow from mice	2.5 × 10^5^ cells	i.v.	(137)
TGFβ1	LPS-induced ALI	Bone marrow from mice	2 × 10^5^ cells	i.t.	(138)
CXCR7	Phosgene-induced ALI	Bone marrow from rats	1 × 10^6^ cells	i.v.	(139)
BPI21/LL-37	CLP-induced sepsis; LPS-induced sepsis	Human umbilical cords	2 × 10^5^ cells	i.v.	(140)
HO-1	LPS-induced ALI	Bone marrow from rats	5 × 10^5^ cells	i.v.	(141)
ACE2^a^	LPS-induced endothelial injury (*in vitro*)	Bone marrow from mice	1 × 10^4^ cells	Co-culture with HPMEC	(142)
AT2R	LPS-induced ALI	Bone marrow from human	1 × 10^6^ cells	i.v.	(145)
CXCR4	LPS-induced ALI	Bone marrow from rats	1 × 10^6^ cells	i.v.	(143)
MIRO-1	Rotenone-induced airway injury	Bone marrow from mice	1 × 10^6^ cells	i.t.	(146)
7ND	Bleomycin-induced ALI	Bone marrow and adipose tissue from mice	5 × 10^5^ cells	i.v.	(148)
HGF	Radiation-induced lung injury	Bone marrow from human	1 × 10^6^ cells	i.v.	(147)
Let-7d	Bleomycin-induced ALI	Bone marrow from human	5 × 10^5^ cells	i.v.	(149)
miR-154	Bleomycin-induced ALI	Bone marrow from human	5 × 10^5^ cells	i.v.	(149)
miR-377-3p	LPS-induced ALI	Human umbilical cords	2 × 10^5^ cells	i.t.	(150)
P130	LPS-induced ALI	Bone marrow from mice	5 × 10^5^ cells	i.t.	(144)
E2F4	LPS-induced ALI	Bone marrow from mice	5 × 10^5^ cells	i.t.	(144)
miR-124-3p	Traumatic ALI	Bone marrow from rats	25 μg exosomes from 2 × 10^7^ MSCs	i.v.	(151)

MSCs, mesenchymal stromal cells; ALI, acute lung injury; LPS, lipopolysaccharide; CLP, cecal ligation and puncture; PBS, phosphate-buffered saline; NS, normal saline; HMPECs, human pulmonary microvascular endothelial cells; i.v., intravenous injection; i.t., intratracheal administration.

a All of the above studies were performed in animal models, except for the in vitro experiment.

## Adverse Effects of MSCs

Despite the promising effects of MSCs in both preclinical and phase 1 clinical trials, the adverse effects of MSC treatment should not be ignored. MSCs are highly adherent to membrane oxygenators, which led to a rapid decline in oxygenator performance during extracorporeal membrane oxygenation (ECMO) in a sheep model of ALI (152, 153). In addition, human MSC administration showed a lack of efficacy in influenza-mediated lung injury in mice (64) and in improving the survival of mice with staphylococcal toxic shock syndrome (154). Senescent MSCs were also unable to show protection in a murine model of LPS-induced lethal endotoxemia (155). In some reports, MSCs were indicated to enhance neutrophil recruitment and its bactericidal functions through TNF-α and IL-1β signaling (156, 157). However, these findings highlight MSCs as a potential risk factor because aberrant neutrophil activation contributes to disease severity and local tissue damage in COVID-19 through the amplification of hypercytokinemia and the formation of neutrophil extracellular traps (NETs) (158). Furthermore, the encouraging findings from animal experiments do not guarantee their efficacy in clinical trials. It was reported that biomarkers of inflammation (IL-6 and IL-8) at 5 days and the length of hospital stay, ventilator-free days, and ICU-free days at 28 days were similar between the MSC treatment and saline placebo groups in a randomized controlled trial of MSCs for ARDS (14).

## Challenges of Future Studies

Clinical trials of MSCs for ARDS and COVID-19 treatment demonstrated safety and tolerance (see **Table 1**). However, both published trials contained very few patients, and much work remains to be undertaken before MSCs can be used in the clinic and even for COVID-19. Firstly, there are various sources of MSCs (including tissues and donors), and the best source needs to be identified. Secondly, the preparation of MSCs (including the culture conditions, passage, and storage) should be standardized and consistent. Thirdly, the best MSC dose, timing, and delivery remain to be further clarified. Finally, the etiologies of sepsis and ARDS vary; thus, optimal treatment with MSCs may depend on different microenvironments. Future studies should take the above suggestions into full consideration and carefully investigate them.

## Conclusions and Perspectives

A growing number of preclinical studies have shown encouraging findings for MSC-based therapy in sepsis and ARDS treatment. Phase 1 clinical trials have also reported that MSCs are safe and well tolerated in ARDS patients. Nevertheless, experimental studies and clinical trials are still needed to fully understand the optimal MSC application in sepsis/ARDS and COVID-19.

## Author Contributions

ZX, YH, and JZ prepared the original draft and reviewed and edited the manuscript. WH, XL, YL, NZ, and LS reviewed and edited the manuscript. XD prepared the tables and figures. All authors contributed to the article and approved the submitted version.

## Funding

The study was funded by the National Natural Science Foundation of China (81870069, 81970071, and 82070084), the Natural Science Foundation of Guangdong Province (2020A1515011459 and 2021A1515012565), the Science and Technology Program of Guangzhou (202102010157), the State Key Laboratory of Respiratory Disease Independent Program (SKLRD-Z-202108), Emergency Key Program of Guangzhou Laboratory (EKPG21-17) and Natural Science Foundation of Guangdong Province (2020A1515011459).

## Conflict of Interest

YL is an editor of Frontiers in Immunology.

The remaining authors declare that the research was conducted in the absence of any commercial or financial relationships that could be construed as a potential conflict of interest.

## Publisher’s Note

All claims expressed in this article are solely those of the authors and do not necessarily represent those of their affiliated organizations, or those of the publisher, the editors and the reviewers. Any product that may be evaluated in this article, or claim that may be made by its manufacturer, is not guaranteed or endorsed by the publisher.
